# Supplementary material for: Red knots in Europe: a dead end host species or a new niche for highly pathogenic avian influenza?
Source: J Gen Virol. 2024 Jul 8;105(7):002003. doi: 10.1099/jgv.0.002003 (PMC11316594; doi:10.1099/jgv.0.002003)
Supplement: Uncited Supplementary Material 1. [file jgv-105-02003-s001.pdf]

Supplementary Figure 1-8:

Maximum likelihood (ML) phylogeny for all 8 gene segments of the HPAI H5N3 virus detected in red knots in the Wadden Sea area in 2020.

Data source: sequence information with virus name, accession and metadata. We acknowledge the listed originating and submitting laboratories for sharing their data

Figure 1: Polymerase Basic 2 (PB2)

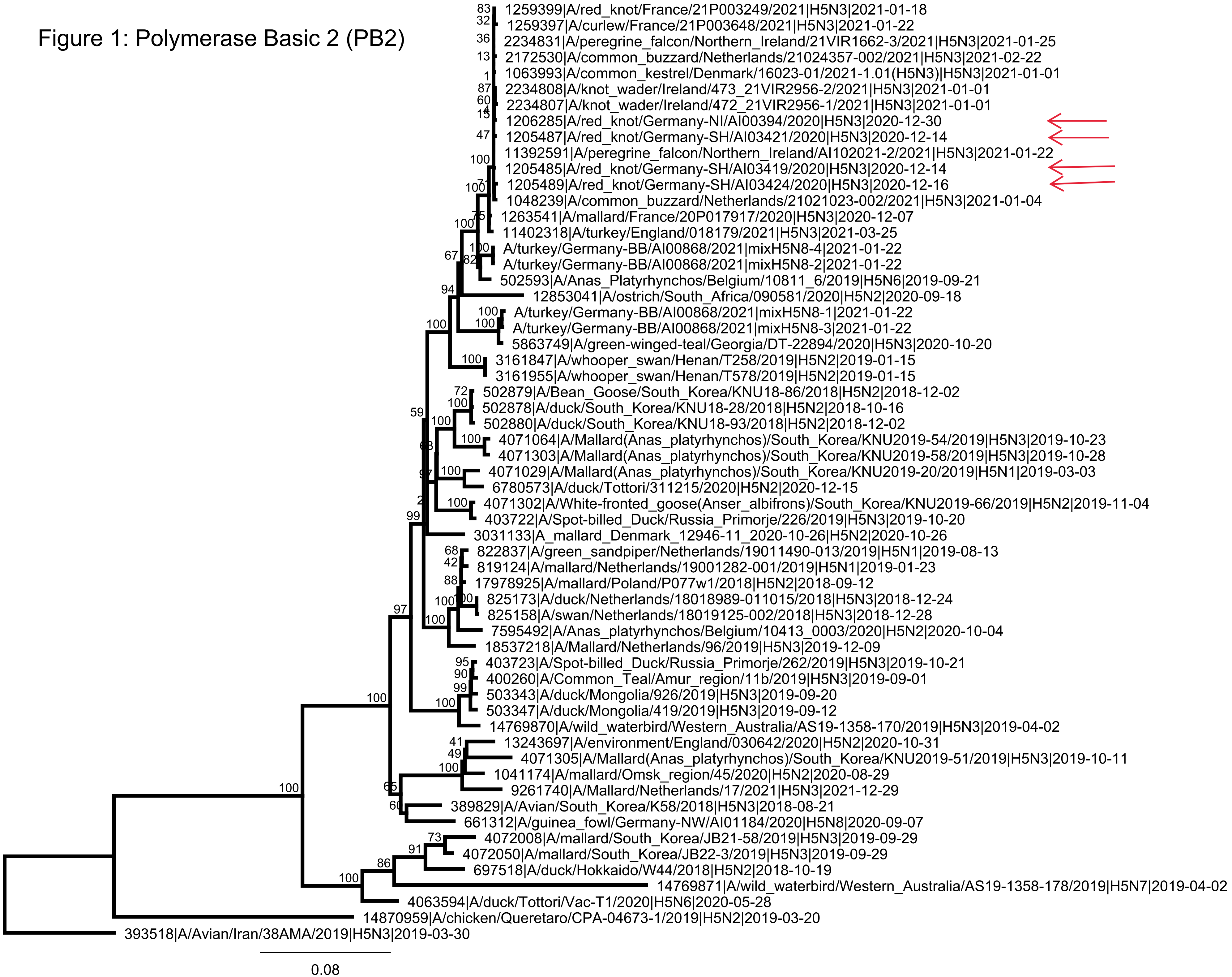

Figure 2: Polymerase Basic 1 (PB1)

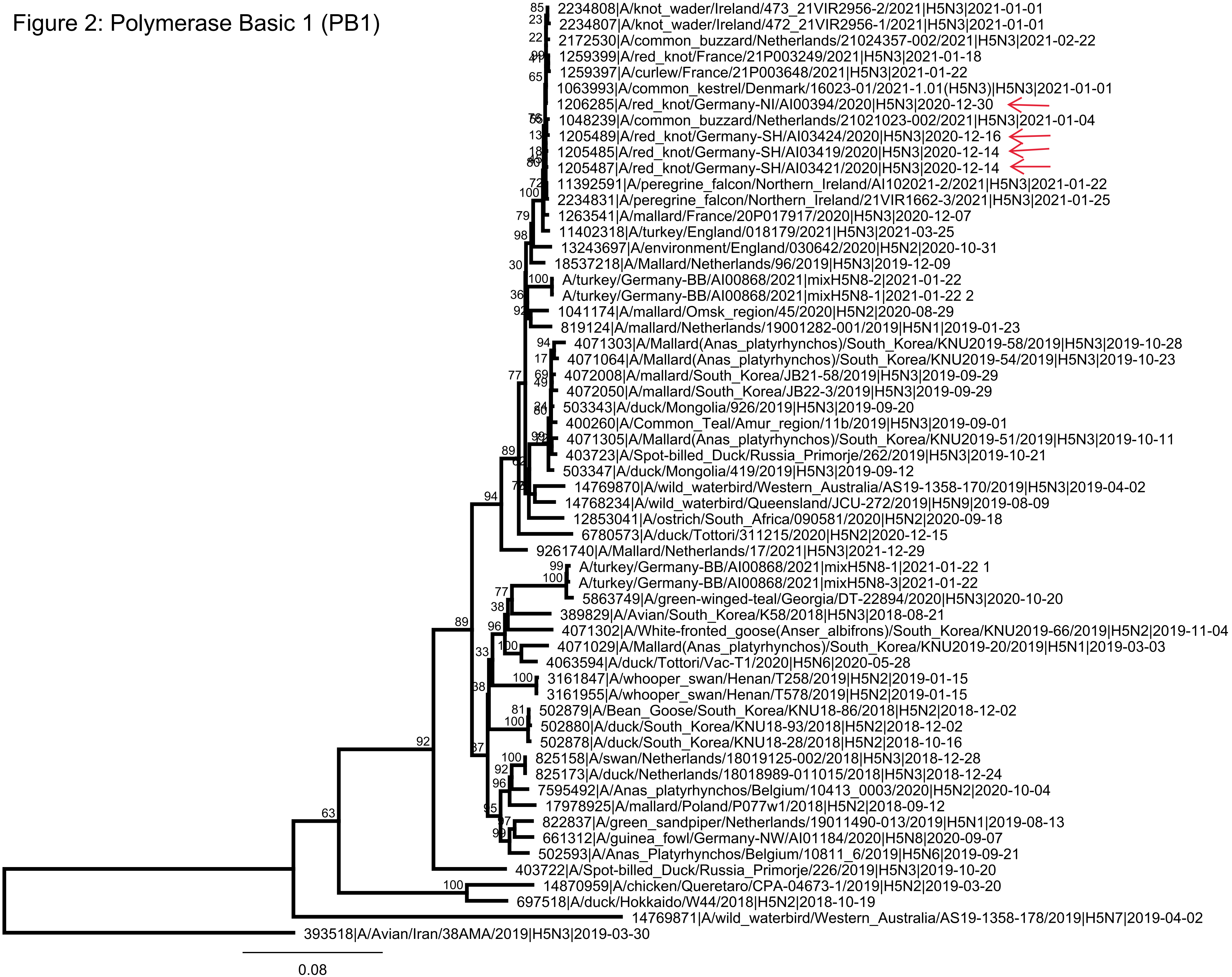

Figure 3: Polymerase Acid (PA)

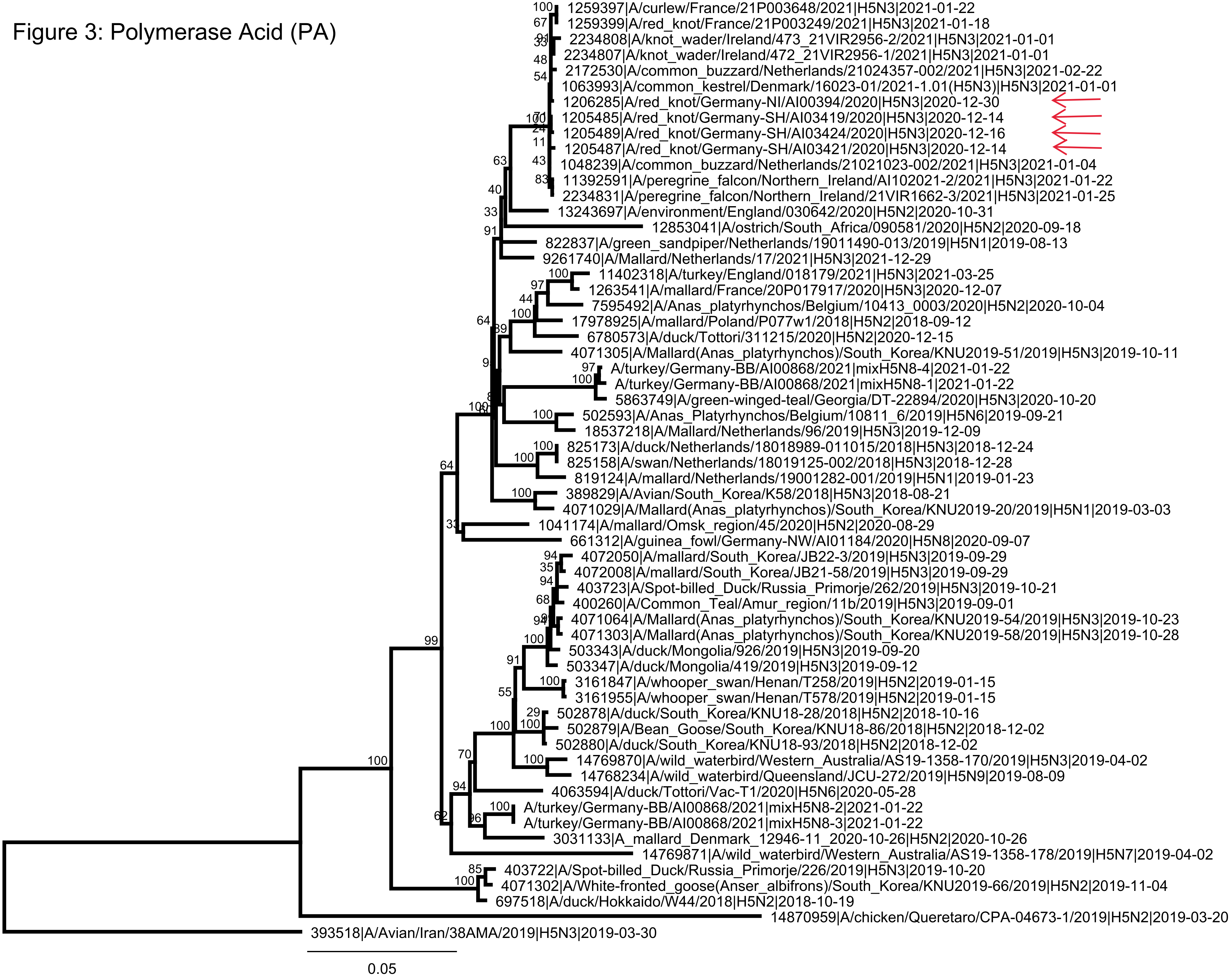

Figure 4:
Haemagglutinin H5

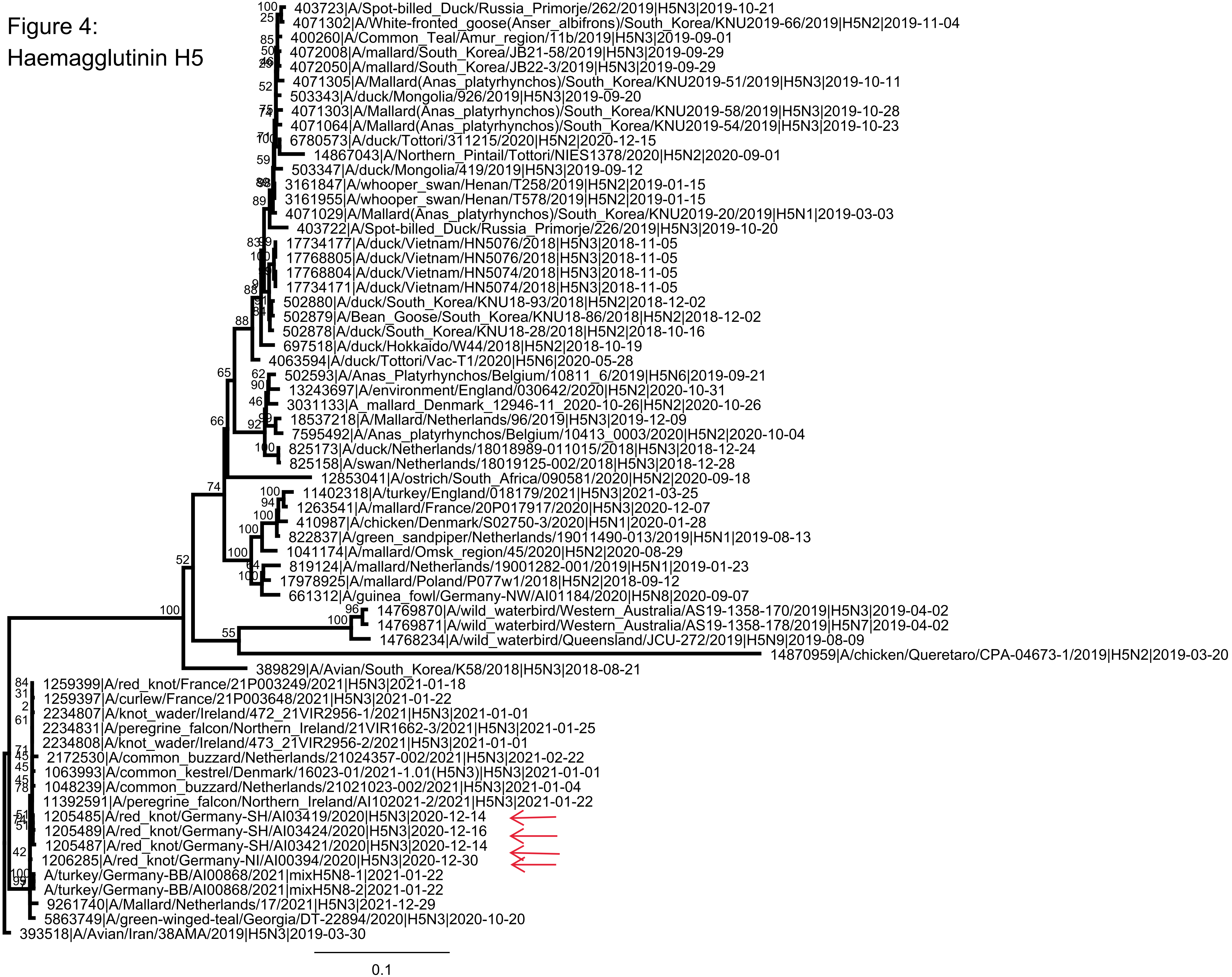

Figure 5: Nucleoprotein (NP)

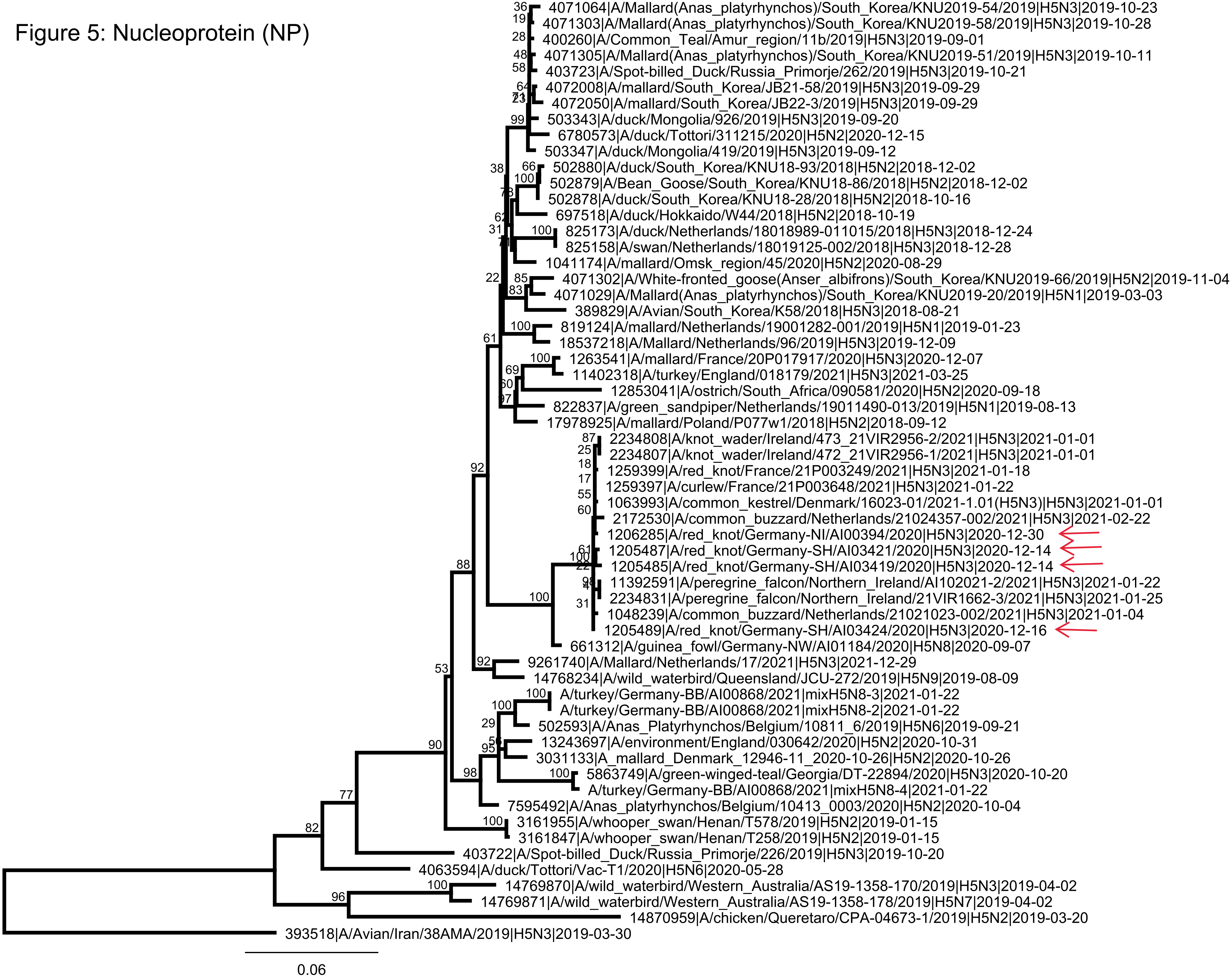

Figure 6: Neuraminidase NA3

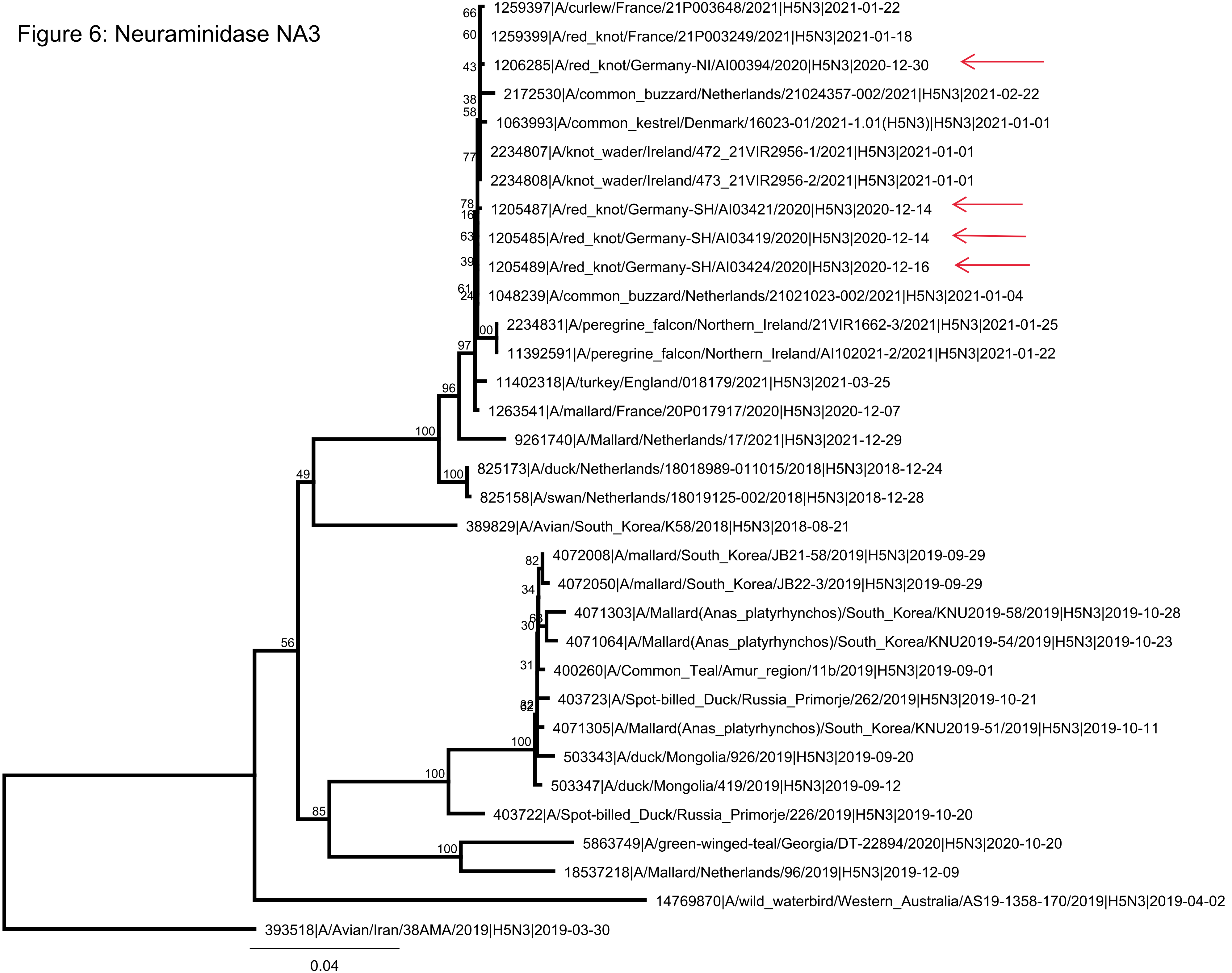

Figure 7: Matrix Protein (MP)

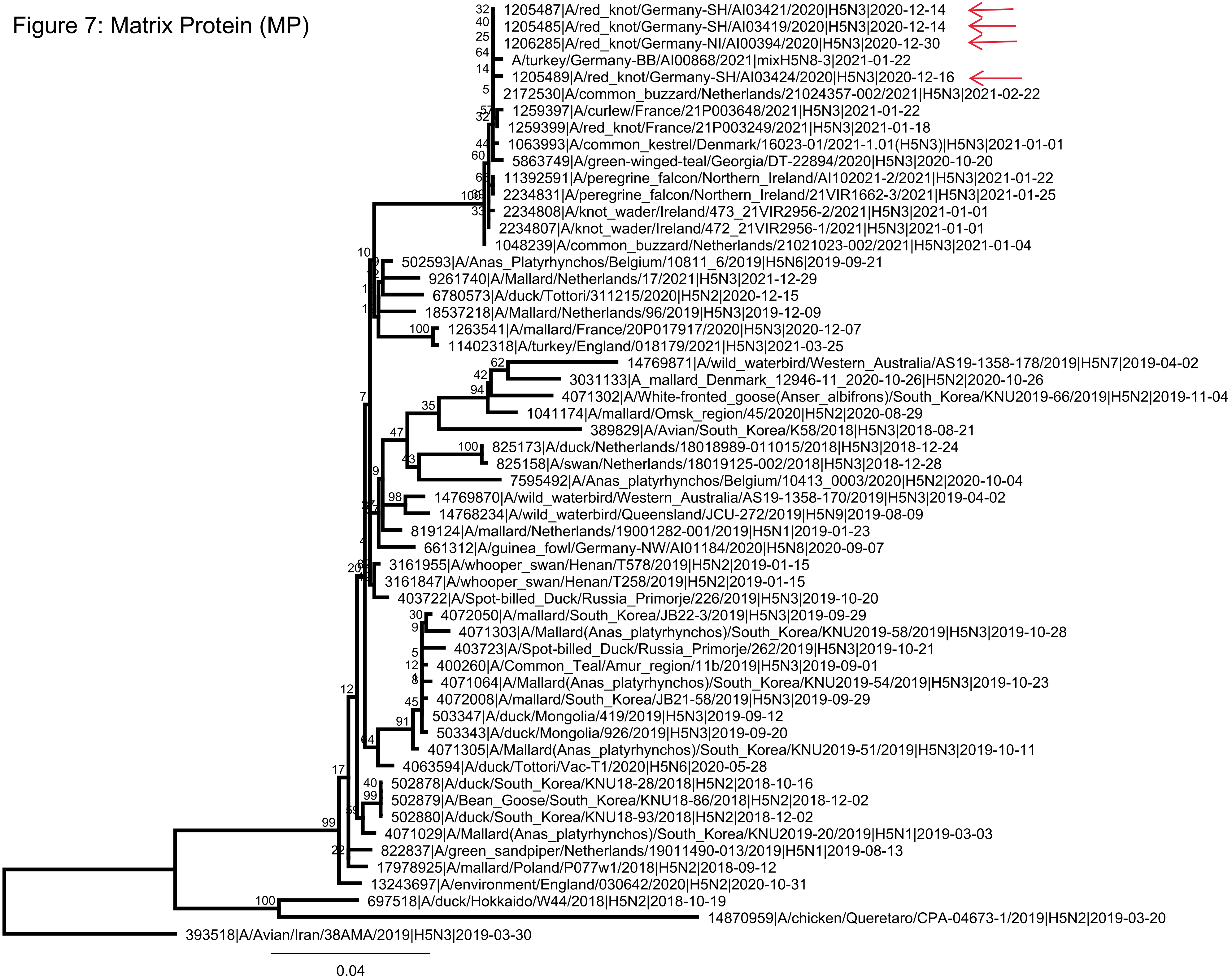

Figure 8:
Non-structural protein (NS)

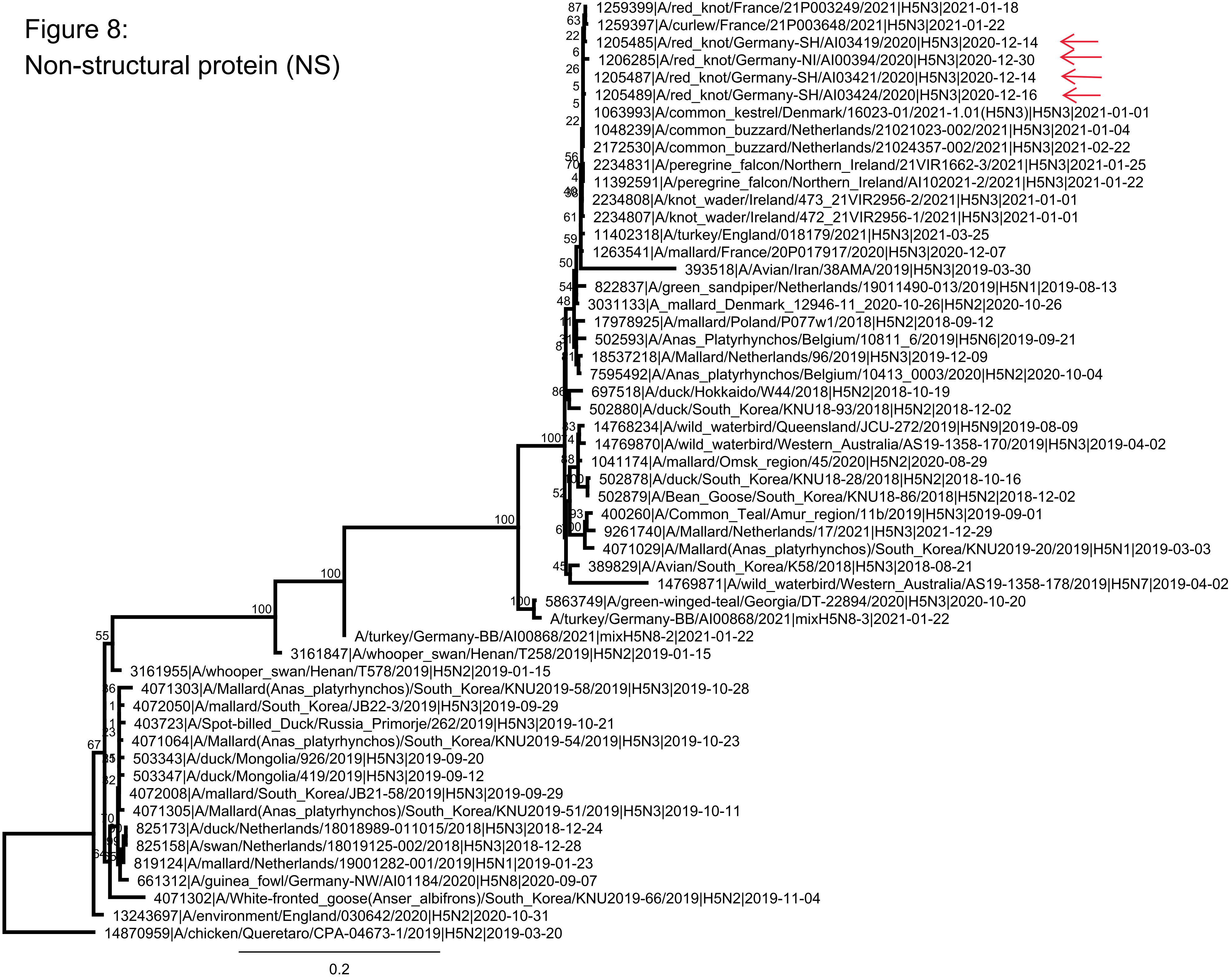

Supplementary Table 1

Data source: sequence information with virus name, accession and metadata. We acknowledge the listed originating and submitting laboratories for sharing their data.

virus name	accession	Subtype	Submitting Lab	Originating Lab
A/Avian/South Korea/K58/2018	389829	H5N3		
A/Avian/Iran/38AMA/2019	393518	H5N3		
A/Common Teal/Amur region/11b/2019	400260	H5N3	National Institute of Animal Health	Research Institute of Experimental and Clinical Medicine
A/Spot-billed Duck/Russia Primorje/226/2019	403722	H5N3	National Institute of Animal Health	Research Institute of Experimental and Clinical Medicine
A/Spot-billed Duck/Russia Primorje/262/2019	403723	H5N3	National Institute of Animal Health	Research Institute of Experimental and Clinical Medicine
A/chicken/Denmark/S02750-3/2020	410987	H5N1	Statens Serum Institute	Statens Serumm Institute
A/Anas Platyrhynchos/Belgium/10811 6/2019	502593	H5N6		
A/duck/South Korea/KNU18-28/2018	502878	H5N2		
A/Bean Goose/South Korea/KNU18-86/2018	502879	H5N2		
A/duck/South Korea/KNU18-93/2018	502880	H5N2		
A/duck/Mongolia/926/2019	503343	H5N3		
A/duck/Mongolia/419/2019	503347	H5N3		
A/guinea fowl/Germany-NW/AI01184/2020	661312	H5N8	Friedrich-Loeffler-Institut	Chemisches und Veterinäruntersuchungsamt Münsterland-Emscher-Lippe
A/duck/Hokkaido/W44/2018	697518	H5N2		
A/mallard/Netherlands/19001282-001/2019	819124	H5N1	Wageningen Bioveterinary Research	Wageningen Bioveterinary Research
A/green sandpiper/Netherlands/19011490-013/2019	822837	H5N1	Wageningen Bioveterinary Research	Wageningen Bioveterinary Research
A/swan/Netherlands/18019125-002/2018	825158	H5N3	Wageningen Bioveterinary Research	Wageningen Bioveterinary Research
A/duck/Netherlands/18018989-011015/2018	825173	H5N3	Wageningen Bioveterinary Research	Wageningen Bioveterinary Research
A/mallard/Omsk region/45/2020	1041174	H5N2	National Institute of Animal Health	Research Institute of Experimental and Clinical Medicine
A/common buzzard/Netherlands/21021023-002/2021	1048239	H5N3	Wageningen Bioveterinary Research	Wageningen Bioveterinary Research
A/common kestrel/Denmark/16023-01/2021-01-01	1063993	H5N3	Statens Serum Institute	Statens Serumm Institute
A/common kestrel/Denmark/16023-01/2021-1.01(H5N3)	1063993	H5N3	Statens Serum Institute	Statens Serumm Institute
A/red knot/Germany-SH/AI03419/2020	1205485	H5N3	Friedrich-Loeffler-Institut	Landeslabor Schleswig-Holstein
A/red knot/Germany-SH/AI03421/2020	1205487	H5N3	Friedrich-Loeffler-Institut	Landeslabor Schleswig-Holstein
A/red knot/Germany-SH/AI03424/2020	1205489	H5N3	Friedrich-Loeffler-Institut	Landeslabor Schleswig-Holstein
A/red knot/Germany-NI/AI00394/2020	1206285	H5N3	Friedrich-Loeffler-Institut	Lebensmittel- und Veterinärinstitut Oldenburg - Standort Veterinärinstitut
A/curlew/France/21P003648/2021	1259397	H5N3	ANSES Agence Nationale De Securite Sanitaire De L'alimentation	Anses (Ploufragan-Plouzané)
A/red knot/France/21P003249/2021	1259399	H5N3	ANSES Agence Nationale De Securite Sanitaire De L'alimentation	Anses (Ploufragan-Plouzané)
A/mallard/France/20P017917/2020	1263541	H5N3	ANSES Agence Nationale De Securite Sanitaire De L'alimentation	Anses (Ploufragan-Plouzané)
A/common buzzard/Netherlands/21024357-002/2021	2172530	H5N3	Wageningen Bioveterinary Research	Wageningen Bioveterinary Research
A/knot wader/Ireland/472 21VIR2956-1/2021	2234807	H5N3	Istituto Zooprofilattico Sperimentale Delle Venezie	Istituto Zooprofilattico Sperimentale delle Venezie, EU/OIE/Reference Laboratory and FAO Reference Centre for AI and ND
A/knot wader/Ireland/473 21VIR2956-2/2021	2234808	H5N3	Istituto Zooprofilattico Sperimentale Delle Venezie	Istituto Zooprofilattico Sperimentale delle Venezie, EU/OIE/Reference Laboratory and FAO Reference Centre for AI and ND
A/peregrine falcon/Northern Ireland/21VIR1662-3/2021	2234831	H5N3	Istituto Zooprofilattico Sperimentale Delle Venezie	Istituto Zooprofilattico Sperimentale delle Venezie, EU/OIE/Reference Laboratory and FAO Reference Centre for AI and ND
A mallard Denmark 12946-11 2020-10-26	3031133	H5N2	Statens Serum Institute	Statens Serumm Institute
A/whooper swan/Henan/T258/2019	3161847	H5N2	Chinese Academy of Forestry	Xi'an Tianlong Science and Technology Co., Ltd.
A/whooper swan/Henan/T578/2019	3161955	H5N2	Chinese Academy of Forestry	Xi'an Tianlong Science and Technology Co., Ltd.
A/duck/Tottori/Vac-T1/2020	4063594	H5N6		
A/Mallard(Anas platyrhynchos)/South Korea/KNU2019-20/2019	4071029	H5N1		
A/Mallard(Anas platyrhynchos)/South Korea/KNU2019-54/2019	4071064	H5N3		
A/White-fronted goose(Anser albifrons)/South Korea/KNU2019-66/2019	4071302	H5N2		
A/Mallard(Anas platyrhynchos)/South Korea/KNU2019-58/2019	4071303	H5N3		
A/Mallard(Anas platyrhynchos)/South Korea/KNU2019-51/2019	4071305	H5N3		
A/mallard/South Korea/JB21-58/2019	4072008	H5N3		
A/mallard/South Korea/JB22-3/2019	4072050	H5N3		
A/turkey/Germany-BB/AI00868/2021	5095644	H5N8	Friedrich-Loeffler-Institut	Landeslabor Berlin-Brandenburg
A/green-winged-teal/Georgia/DT-22894/2020	5863749	H5N3	Royal Veterinary College	Erasmus Medical Center
A/duck/Tottori/311215/2020	6780573	H5N2		
A/Anas platyrhynchos/Belgium/10413 0003/2020	7595492	H5N2	Sciensano, Department of Animal Infectious Diseases	Sciensano - Animal Infectious Diseases
A/Mallard/Netherlands/17/2021	9261740	H5N3	Erasmus Medical Center	Erasmus Medical Center
A/peregrine falcon/Northern Ireland/AI102021-2/2021	11392591	H5N3	Animal and Plant Health Agency (APHA)	Animal and Plant Health Agency (APHA)
A/turkey/England/018179/2021	11402318	H5N3	Animal and Plant Health Agency (APHA)	Animal and Plant Health Agency (APHA)
A/ostrich/South Africa/090581/2020	12853041	H5N2	University of Pretoria	Western Cape Provincial Veterinary Laboratory
A/environment/England/030642/2020	13243697	H5N2	Animal and Plant Health Agency (APHA)	Animal and Plant Health Agency (APHA)
A/wild waterbird/Queensland/JCU-272/2019	14768234	H5N9		
A/wild waterbird/Western Australia/AS19-1358-170/2019	14769870	H5N3		
A/wild waterbird/Western Australia/AS19-1358-178/2019	14769871	H5N7		
A/Northern Pintail/Tottori/NIES1378/2020	14867043	H5N2		
A/chicken/Queretaro/CPA-04673-1/2019	14870959	H5N2		
A/duck/Vietnam/HN5074/2018	17734171	H5N3		
A/duck/Vietnam/HN5076/2018	17734177	H5N3		
A/duck/Vietnam/HN5074/2018	17768804	H5N3		Center for Research on Influenza Pathogenesis
A/duck/Vietnam/HN5076/2018	17768805	H5N3		Center for Research on Influenza Pathogenesis
A/mallard/Poland/P077w1/2018	17978925	H5N2	National Veterinary Research Institute	National Veterinary Research Institut Poland, PIWet-PIB
A/Mallard/Netherlands/96/2019	18537218	H5N3	Erasmus Medical Center	Erasmus Medical Center
